# Production and Characterisation of an Exopolysaccharide by *Vreelandella titanicae* Zn11_249 Isolated from Salar de Uyuni (Bolivia)

**DOI:** 10.3390/polym17172362

**Published:** 2025-08-30

**Authors:** Esteban Sabroso, José M. Martínez, Enrique Sánchez-León, Nuria Rodríguez, Ricardo Amils, Concepción Abrusci

**Affiliations:** 1 Department of Molecular Biology, Faculty of Sciences, Autonomous University of Madrid (UAM), Cantoblanco, 28049 Madrid, Spain; 2 Molecular Ecology of Extreme Environments, Scientific Program Interactions with the Environment, Centro de Biología Molecular Severo Ochoa (UAM-CSIC), 28049 Madrid, Spain; 3 Centro de Astrobiología (CAB), CSIC-INTA, Carretera de Ajalvir km 4, Torrejón de Ardoz, 28850 Madrid, Spain

**Keywords:** Salar de Uyuni, chao-kosmotropic, exopolysaccharides, *Vreelandella*, characterisation, antioxidant, cytotoxicity

## Abstract

The extremophilic strain *Vreelandella titanicae* Zn11_249 was isolated from Salar de Uyuni, an environment with high salinity, among other extreme factors. This study researched the optimised production, characterisation, antioxidant activity, and cytotoxicity of exopolysaccharides (EPS) produced by this strain under different ionic stresses. Zn11_249 was cultured in a minimal medium with glucose as the sole carbon source as a control, and under kosmotropic (NaCl, 1 M) and chaotropic (LiCl, 0.3 M) conditions, yielding EPS_U1_, EPS_U2_, and EPS_U3_, respectively. Maximum EPS production (336 mg/L) occurred under chaotropic conditions after 96 h. EPSs were characterised using the following techniques: Gas chromatography (GC-MS); Attenuated Total Reflectance Fourier Transform Infrared Spectroscopy (ATR-FTIR); Thermogravimetric Analysis (TGA); and Differential Scanning Calorimetry, (DSC). The results showed differences between the molecular weights for EPS_U1_ (3.9 × 10^4^ Da), EPS_U2_ (3.9 × 10^4^ Da), and EPS_U3_ (5.85 × 10^4^ Da). Their monosaccharide molar ratios (%) were 40/25/25/10 in EPS_U1_, 10/30/30/30 in EPS_U2_, and 25/25/25/25 in EPS_U3_, composed of mannose, galactose, rhamnose, and glucose, respectively. Functional group analysis confirmed their heteropolysaccharide nature. Thermal profiles suggest the potential of these exopolysaccharides as biomaterials. Antioxidant tests demonstrated significant activity against DPPH, OH, and O_2_ radicals, while cytotoxicity assays showed no toxicity. These results highlight the biotechnological potential of EPSs from *Veelandella titanicae* Zn11_249 for biomedical and cosmetic uses.

## 1. Introduction

Exopolysaccharides (EPSs) are secreted by microorganisms in response to specific environmental conditions and play a key role in cell protection, biofilm formation, and adaptation to extreme environments [1,2]. Various industries such as the food, pharmaceutical, and environmental sectors have focused on these compounds, as they are environmentally friendly and an inexhaustible source of this resource [3].

Currently, interest is focused on the search for extremophilic EPS-producing bacteria. These extreme environments are underexplored and are expected to provide various biotechnological applications. Among these extremophiles, the EPSs of halophilic and halotolerant bacteria have been the subject of recent interest due to their protective role in high-salinity conditions [2]. Halotolerant bacteria can grow at concentrations below 0.2 M and with high salt concentrations. These microorganisms are recognised as EPS producers, with extensive industrial applications [1,4,5].

The most common genera of this group producing EPSs are *Alteromonas*, *Bacillus*, *Chromohalobacter*, *Halomonas*, and *Vibrio*. The EPSs obtained are commonly heteropolysaccharides, whose most common monomers are mannose, glucose, galacturonic acid, or galactose [6]. These EPSs produced by salt-tolerant bacteria have a higher water-holding capacity, stability in hypersaline environments, and a strong interaction with metal ions. These are interesting features for biotechnological applications [7].

Among the genera to highlight, there is the genus *Halomonas*, from the recently reclassified family Halomonadaceae [8]. This genus represents a wide variety of halotolerant species capable of secreting EPSs with environmental and health potential. From the environmental aspect, there are the EPSs of *Halomonas nitroreducens* WB1 and *Halomonas almeriensis* M8T with heavy metal adsorption capacity and pseudoplastic properties [7,9]. *Halomonas stenophila* HK30 and *Halomonas elongata* S6 produce EPSs with emulsifying and flocculant activity [10,11]. For biomedical and cosmetic uses, *Halomonas smyrnensis* K2 produced an EPS with antioxidant and antibiofilm activity [12], and *Halomonas smyrnensis* AAD6T and the EPS secreted by *Halomonas stenophila* B100 presented antitumor activity [13,14].

Thus, the search for new halotolerant strains of the genus *Halomonas* is very interesting due to its biotechnological potential. Salar de Uyuni (SdU) is an environment with microorganisms that have not yet been researched in depth. It represents an interesting extremophile environment for the study of these halotolerant bacteria. This salt flat is located in the southeastern region of Potosí, in the Bolivian Antiplano depression [15]. It is the largest athalosaline ecosystem in the world (10,582 km^2^) and is located at an altitude of 3653 m above sea level. It has truly unique characteristics such as kosmotropic solutes (such as NaCl) and chaotropic solutes (such as LiCl, MgCl_2_, and CaCl_2_). These conditions influence the biological processes of the halotolerant bacteria that inhabit the salt flat [16,17], making this environment very interesting for the search for new EPS-producing bacteria with biotechnological potential [18].

The hypothesis of this work is that the three different conditions found in the Salar de Uyuni (control, kosmotropic, and chaotropic) can influence both the production of EPSs by *Vreelandella titanicae* Zn11_249 and their potential biotechnological applications, when compared to classical growth conditions.

This study was conducted using *V. titanicae* Zn11_249, formerly *Halomonas titanicae* [8], a Gram-negative, heterotrophic, aerobic, non-spore-forming, motile bacterium with peritrichous flagella [19]. The objectives of this work were to evaluate EPS production, estimate their molecular weight, and characterise them under both chaotropic and kosmotropic conditions. Additionally, biosynthetic pathways, antioxidant activity, and cytotoxicity were analysed to further explore their potential biotechnological applications.

Here, we report EPS production by *V. titanicae* Zn11_249 under control, kosmotropic, and chaotropic conditions. To our knowledge, this is the first description of exopolysaccharide production by a bacterium thriving in a chao-kosmotropic environment. Additionally, the antioxidant properties of the EPSs were evaluated, highlighting their potential relevance in biomedical, cosmetic, and food-related applications.

## 2. Materials and Methods

### 2.1. Chemicals and Standards

Glucose, trypticase soy agar (TSA), dextran molecular weight standards (5, 12, 50, and 80 kDa), 1,1-diphenyl-2-picryl-hydrazyl radical (DPPH), hydrogen peroxide (H_2_O_2_), salicylic acid, sodium dodecyl sulphate (SDS), pyrogallol, ferrous sulphate (FeSO_4_), hydrochloric acid (HCl), L-ascorbic acid (Av), foetal bovine serum (FBS), L-glutamine, penicillin (100 IU/mL), streptomycin, and Dulbecco’s Modified Eagle Medium (H-DMEM) were purchased from Sigma-Aldrich (St. Louis, MO, USA). The HeLa cell line (human cervix adenocarcinoma) was kindly provided by the Centro de Biología Molecular Severo Ochoa (CBM-UAM, Universidad Autónoma de Madrid, Madrid, Spain) in Sigma-Chemical (St Louis, MO, USA). The MTT reagent [3-(4,5-dimethylthiazol-2-yl)-2,5-diphenyltetrazolium bromide] was purchased from GE Healthcare (Uppsala, Sweden). Sephadex G-100 and DEAE-52 anion exchange resin were acquired from Aldrich Chemical Company, Inc. (Milwaukee, WI, USA). Trifluoroacetic acid (TFA) was obtained from Aldrich^®^ (Schnelldorf, Germany).

### 2.2. Bacterial Strain Sample

The strain *Vreelandella titanicae* Zn11_249 was isolated in Marine Agar (Condalab, Madrid, Spain) from a brine sample collected from the ojos del Salar in the non-industrial zone (E-W transect, latitude 20°10′23.3″ S and longitude 67°43′20.7″ W ) of SdU during the dry–cold season (June 2013). Sample characterisation was performed as described in Martínezet al. 2021 [18]. Halite is the main mineral, along with the presence of gypsum accumulations. Elemental analysis showed its having the following chemical composition 0.001 g/L K^+^, 294.48 g/L Na^+^, 1.28 g/L Cl^-^, 0.009 g/L S, 0.345 g/L Mg^2+^, 0.01 g/L Li^+^, 0.01 g/L Ca^2+^, and 0.0002 g/L Br^-^ as the main ions. The water activity (a_w_) was 0.728.

### 2.3. Analysis of Bacterial Growth, Colony Forming Units (CFU)/mL, pH, and EPS Production

Measures of bacterial growth and EPS production were carried out using minimal medium U_1_ as a control: Na_2_HPO_4_, 0.05 M; KH_2_PO_4_, 0.02 M; NaCl, 0.01 M; NH_4_Cl, 0.02 M; MgSO_4_ × 7H_2_O, 0.01 M; CaCl_2_, 0.001 M. Glucose (0.02 M) was selected as the sole carbon source due to its high industrial applicability and low cost, as well as its subsequent correct correlation in the genomic analysis [20]. The comparative analysis of EPS production in kosmo-chaotropic conditions was performed by modifying the U_1_ medium by increasing the content of the kosmotropic salt NaCl to 1 M (U_2_). As a representative of chaotropicity, the NaCl present in the U_1_ medium was replaced by the chaotropic salt LiCl at 0.3 M (U_3_). In previous analyses for this study, the optimal growth conditions for the strain in LB Broth (Sigma-Aldrich, St. Louis, MO, USA) were found to be above 1.5 M NaCl and 0.3–0.4 M LiCl. For this study, these salt concentrations were carefully selected to ensure rapid and proper bacterial growth while avoiding high salt levels that could interfere with polymer characterisation, even though they did not represent the optimal growth conditions of each medium.

These conditions were established by transferring the strain, previously inoculated in TSA medium and incubated at 30 °C for 72 h, to a 250 mL flask filled with 50 mL of the different growth cultures.

The inoculum was prepared at an optical density measured at 600 nm with a spectrophotometer Biowave II^+^ (Biochrom, Cambridge, UK). In order to determine when the culture reached the beginning of its stationary phase, samples were measured at 24, 48, 72, 96, and 120 h of incubation. The cell growth number was evaluated by different plating dilutions (10^−3^–10^−6^) incubated at 30 °C for 168 h with TSA agar medium (OD_600_ = 1 Abs). A Thermo Orion pH Meter Model 2Star (ThermoScientific, Asheville, NC, USA) was used to determine the pH values during the experiment.

For the determination of EPS production, the strain was inoculated from the stock culture in TSA medium and incubated at 30 °C for 24 h. After that, the strains were transferred into flasks of 250 mL filled with 50 mL of different mediums (U_1_, U_2_, and U_3_) using 0.5 mL of inoculum (OD_600_ = 1 Abs/2.0 × 10^7^ CFU/mL). The flasks were incubated in a rotary shaker incubator (model Orbitek LJEIL; Scigenics Biotech, Bangalore, India) at 30 °C and 130 rpm for 24 h. After the first incubation, 10 mL of this broth (OD_600_ = 1 Abs) was inoculated into flasks of 2000 mL filled with 1000 mL of each medium. The flasks were incubated at 30 °C and 130 rpm for 96 h, when the stationary phase was reached. Three independent assays were performed in U_1_, U_2_, and U_3_. The EPSs were isolated and assessed for each treatment.

### 2.4. Scanning Electron Microscopy (SEM) in Three Different Media

Samples from cultures grown in the three media (U_1_, U_2_, U_3_) at 96 h were filtered through 0.22 μm membranes and fixed with 20% glutaraldehyde for 2 h to preserve cell structure and maintain surface integrity. Three washes were then performed using distilled water and dehydrated using increasing concentrations of ethanol: 30, 50, and 70% for 20 min, 90% for 2 × 20 min, and 100% for 2 × 30 min at room temperature. Standard gold plating was performed to prevent charge accumulation and improve image quality [21]. The samples were observed by scanning electron microscopy using a JEOL-5600 microscope coupled with Oxford INCA X-sight EDAX Energy Dispersive X-ray Microanalysis.

### 2.5. Isolation, Purification, and Molecular Weight of EPS in Three Different Media

The isolation and purification of each EPS obtained from each medium were performed following the method of Sánchez-León et al. [22]. The cultures obtained from the strain were centrifuged at 13,154× *g* for 30 min at 4 °C with a DuPont Sorvall RC−5 centrifuge (Wilmington, DE, USA). The EPSs were precipitated from the supernatant with cold ethanol 96% (three times the volume) and left overnight at 4 °C. The precipitates were collected by centrifugation at 13,154× *g* for 30 min at 4 °C and dissolved in Milli-Q water. Then, the crude EPSs were dialysed at 4 °C with Milli-Q water for 48 h. The dialysed contents were freeze-dried by lyophilisation with a Flexy–Dry MPTM freeze dryer (FtS Systems Inc., Stone Ridge, NY, USA) for 48 h, and the dry weights of the powdered EPSs were determined. For the purification of the EPSs, the products (10 mL, 10 mg/mL) were subjected to a DEAE−52 anion exchange column (2.6 × 30 cm) and eluted with deionised water. Concentrations of 0.2, 0.5, 1.0, and 1.5 M of NaCl were used as eluent at 1 mL/min flow rate. The eluents (10 mL/tube) were monitored by the phenol–sulphuric acid method [23], and the carbohydrate-positive fractions were pooled, concentrated, and lyophilised. The obtained EPSs were named as follows according to the medium used: EPS_U1_, EPS_U2_, and EPS_U3_.

### 2.6. Molecular Weight, Compositional Analysis, and Characterisation of the EPSs in Three Different Media

#### 2.6.1. Molecular Weight

Molecular weights of the purified fraction from each EPS were determined by gel filtration chromatography [24]. Standard reference dextrans of 5, 12, 50, and 80 KDa weight were used. A Sephadex G−100 column (1.6 × 50 cm) eluting with 0.2 mol^−1^ NaCl solution at a flow rate of 1 mL/min was used for this experiment. The molecular weights of EPS_U1_, EPS_U2_, and EPS_U3_ were derived from the standard plot of the reference dextrans (R^2^ = 0.9942).

#### 2.6.2. Compositional Analysis

To determine the monosaccharide composition prepared as described in the literature [22], EPS_U1_, EPS_U2_, and EPS_U3_ were hydrolysed with 0.5 M of trifluoroacetic acid (TFA) at 120 °C for 2 h. Samples were treated before and after the process with N_2_. The derivative products were used for the determination of the monosaccharide composition by gas chromatography coupled with mass spectrometry detector (GC–MS). An EVOQ GC–TQ Bruker (Bruker, Billerica, MA, USA) gas chromatography system was employed. A total of 1 µL of samples was injected in the ratio of 100:1 in split-less mode with source temperature at 230 °C. The separation was held in an Rxi^®^−5Sil MS (Restek Corporation, Bellefonte, PE, USA) capillary column having a 30 m length × 0.250 mm width and 0.25 µm, with helium as a carrier gas at a constant flow rate of 1 mL/min. The initial temperature was 50 °C with a hold time of 2 min and increase to 280 °C by 10 °C ramp increments with a 5 min hold time. Monosaccharides such as glucose, arabinose, xylose, mannose, galactose, fructose, galacturonic acid, and glucuronic acid were used as standards.

#### 2.6.3. Attenuated Total Reflectance/FT-Infrared Spectroscopy (ATR-FTIR)

The EPS_U1_, EPS_U2_, and EPS_U3_ structural–functional groups were detected using attenuated total reflectance/FT–infrared spectroscopy (ATR–FTIR). IR spectra were obtained using a BX–FTIR spectrometer (Perkin Elmer, Waltham, MA, USA) coupled with an ATR accessory, MIRacleTM –ATR (Pike Technologies, Cottonwood, AZ, USA), and spectra were obtained from 32 scans at 4 cm^–1^ of resolution from 400 to 4000 cm^–1^.

#### 2.6.4. Differential Scanning Calorimetry (DSC) Analysis

Differential scanning calorimetry (DSC) was performed on DSC Q100 (TA Instruments, New Castle, DE, USA). The calorimeter was previously calibrated and certified by the National Institute of Standards and Technology NIST. A total of 0.5–2 mg of dried EPSt sample was placed in an aluminium pan without a lid. Then, it was analysed using an empty pan as a reference and 50 mL/min air purge gas. The heating rate was 10 °C/min from 20 °C to 600 °C.

### 2.7. PCR Amplification and GENOMIC Analysis of EPSs Synthesis.

DNA extraction was performed using the CTAB-phenol-chloroform protocol [25]. DNA concentration was analysed by fluorometry (Qubit v2.0, Invitrogen, Waltham, MA, USA), and its integrity was verified using a 1% (*w*/*v*) agarose gel diluted in 0.5× TBE (70 V for 20 min). The DNA was kept at −20 °C.

Isolate identification was performed by amplifying the 16S rRNA gene as described by García et al. (2018) [25]. Reads were edited and assembled using MEGA software (version 11) [26].

Bioinformatic profiling of the genes involved in EPS biosynthesis in *V. titanicae* Zn11_249 was carried out using the assembled genome deposited by us in ENA/NCBI (CAYELD010000000/PRJEB81508). Genes potentially involved in the biosynthesis of sugars nucleotides, glycosyltransferases, and EPS transport systems were predicted using the PROKKA v1.14.6 [27] software using the default parameters and RAST [28] using the *V*. *titanicae* BH1 genome as a reference (NCBI accession number: PRJNA169611) [29].

### 2.8. Antioxidant Activities of the EPSs in Three Different Media

The free radical scavenging activities for the 1,1–diphenyl–2–picryl–hydrazyl radical (•DPPH), hydroxyl radical (•OH^−^), and superoxide anion (O_2_^−^•) were assessed as indicators of the antioxidant activity of the EPSs. Absorbances were measured using a FLUOstar Omega BMG LABTECH (Aylesbury, UK) spectrophotometer with MARS Data Analysis Software (v5.7) for DPPH• (OD_525_), •OH^-^ (OD_510_), and O_2_^−^• (OD_325_) and using L-ascorbic acid (Av) as a control. The purified EPSs were easily soluble in Mili-Q water at room temperature for analysis.

#### 2.8.1. DPPH Free Radical Scavenging Activity

DPPH scavenging activity was assessed using the procedure described by Niknezhad et al. (2018) [30]. The reaction mixture contained 50 µL of EPS at different concentrations (from 0.1 to 10 mg/mL) and 100 µM of DPPH-ethanolic solution (96 °C) (Sigma Chemical, St Louis, MO, USA). The mixtures were shaken and incubated in the dark at 25 °C. After 30 min, the absorbance was recorded, and the data were treated with formula (1) to evaluate the percentage [%] of radical scavenging activity of •DPPH; A_0_ refers to the reaction mixture with DPPH and without purified EPS (2); A_2_ refers to the unreacted mixture (3); A_1_ is represented as a reaction mixture (4), and these are shown as follows. The procedure was carried out in the same way for each of the three EPSs obtained, EPS_U1_, EPS_U2,_ and EPS_U3_:

DPPH scavenging activity = [1 − (A_1_ − A_2_)/A_0_] × 100 (1)

A_0_ = 50 µL Milli Q water + 100 µL ethanol 96% (2)

A_2_ = 50 µL Milli Q water + 100 µL de DPPH − ethanol solution (3)

A_1_ = 50 µL (EPS or Av) + 100 µL de DPPH − ethanol solution (4)

#### 2.8.2. Hydroxyl Radical Scavenging Activity

The described FeSO_4_-salicylic acid method described by Sun et al. (2015) [31] was used to determine the hydroxyl radical scavenging activity of the EPSs. The reaction mixtures were prepared with 40 µL of an FeSO_4_ solution (9 mM), 40 µL of an ethanol solution–salicylic acid solution (9 mM), and 40 µL of the different concentrations of the EPSs. Finally, 40 µL of H_2_O_2_ (8.8 mM) was used to initiate the reaction. The mixtures were incubated at 37 °C, measuring the absorbance after 30 min. Formula (5) was used to evaluate the percentage [%] of •OH^−^ scavenging activity; A_0_ refers to the reaction mixture with salicylic acid and without purified EPS (6); A_1_ is represented as the reaction mixture (7); A_2_ refers to the mixture without salicylic acid (8), and these are shown below. The procedure was carried out in the same way for each of the three EPSs obtained, EPS_U1_, EPS_U2,_ and EPS_U3_:

Hydroxyl radical scavenging activity = [1 − (A_1_ − A_2_)/A_0_] × 100 (5)

A_0_ = 40 µL Milli Q water + 40 µL FeSO_4_ solution + 40 µL ethanol 96% + 40 µL H_2_O_2_ (6)

A_2_ = 40 µL Milli Q water + 40 µL FeSO_4_ solution + 40 µL ethanol − salicylic solution + 40 µL of H_2_O_2_ (7)

A_1_ = 40 µL (EPS or Av) + 40 µL FeSO_4_ solution + 40 µL ethanol − salicylic solution + 40 µL H_2_O_2_ (8)

#### 2.8.3. Superoxide Anion Scavenging Activity

The superoxide scavenging activity was assessed using the method described by Balakrishnan et al. (2011) [32]. The reaction mixture contained 0.3 mL of the different concentrations of the EPSs mixed with 2.5 mL of phosphate buffer (50 mM, pH 8) and 90 µL of pyrogallol (3 mM) dissolved in HCl solution (10 mM). The mixture was incubated at 25 °C, and the absorbance was measured from 0 min to 10 min. Formula (9) was used to evaluate the percentage [%] of O_2_−• scavenging activity; A_0_ and A_10_ represent the reaction mixture at 0 and 10 min, respectively (10); C_0_ and C_10_ represent the reaction mixture without pyrogallol at 0 and 10 min, respectively (11), and these are shown as follows. The procedure was carried out in the same way for each of the three EPSs obtained, EPS_U1_, EPS_U2,_ and EPS_U3_:

Superoxide scavenging activity = 1 − [(A_10_/C_10_) − (A_0_/C_0_)] × 100 (9)

A_0_ and A_10_ (0 min and 10 min) = 0.3 mL (EPS or Av) + 2.6 mL fosfate Buffer + 90 mL pyrogallol − HCl (10)

C_0_ and C_10_ (0 min and 10 min) = 0.3 mL Milli Q water + 2.6 mL fosfate Buffer + 90 mL pyrogallol − HCl (11)

### 2.9. Biocompatibility Studies in Three Different Media

#### 2.9.1. Culture of Cells

The reference cell line for the study of EPS biocompatibility was HeLa cells (human T-cell lymphoblast-like cell line). Cells were cultured in Dulbecco’s modified Eagle’s medium (H-DMEM) supplemented with 10% Foetal Bovine Serum (FBS) and 2 mM of L-glutamine, penicillin (100 IU/mL), and streptomycin (100 mg/mL). The cultures were maintained in a 5% CO_2_ atmosphere at 37 °C during the different experiments [33].

#### 2.9.2. Cytotoxicity Assay

Cell viability was assessed by the reduction in the MTT reagent (3–[4,5–dimethyl-thiazol−2–yl] −2,5–diphenyltetrazoliumbromide) to formazan following the method proposed by Pérez-Blanco et al. (2022) and Morro et al. (2017) [34,35]. HeLa cells were seeded in a 24-well culture plate (5 × 10^5^ CFU/mL), and 100 µL of different concentrations of EPSs (0, 25; 50; 100; 200; 400; 800; 1000; 1500; 2000 y 2500 μg/mL) was transferred into each well for 24 h of treatment. The purified EPSs were easily soluble in Mili-Q water at room temperature for the preparation of the concentrations. The absorbance at OD_590_ was measured using a microplate reader (LT−4000, Labtech International Ltd., Lewes, UK). Determination of the percent protection of the EPSs on HeLa Cells against oxidative stress [%] was calculated according to Equation (12), where A_1_ represents the absorbance of HeLa cells treated with H_2_O_2_ and the MTT solution, and A_2_ represents the absorbance of HeLa cells that were not subjected to any treatment with the MTT solution. The procedure was carried out in the same way for each of the three EPSs obtained, EPS_U1_, EPS_U2_, and EPS_U3_, as follows:

Cell viability = (A_1_/A_2_) × 100 (12)

The statistical differences of the different EPSs and their concentrations were used to determine the cell viability compared to a control without EPSs, together with the cytotoxicity limit established according to ISO 10993-5:2009 (UNE-EN ISO 10993-5:2009, International Standardisation Organisation) [36].

### 2.10. Determination of the Antioxidant Ability at the Cellular Level in Three Different Media

#### Establishment of Injury Model

The establishment of the injury model against HeLa cells was assayed following the method described by Huang-Lin et al. (2022) [33]. The HeLa cell density that was used was 5 × 10^5^ CFU/well and was seeded in 96-well plates for 24 h. After this time, the solution was removed, and 100 µL of different concentrations of H_2_O_2_ (0.25, 0.5, 1, and 2 mM) were added for 1 h under 5% CO_2_ atmosphere incubated at 37 °C. After the exposure time, the solution was removed, and new medium was added to the plates to determine cell viability by the MTT method described in Section 2.9.2. The procedure was carried out in the same way for each of the three EPSs obtained, EPS_U1_, EPS_U2_, and EPS_U3_.

### 2.11. Statistical Analysis

All experiments were performed in triplicate. An analysis of variance test (ANOVA) was performed to make statistical comparisons by using the Statistical Package for the Social Sciences version 21 (SPSS^®^ Inc., Chicago, IL, USA). *p* < 0.05 was considered statistically significant.

## 3. Results and Discussion

### 3.1. Strain Identification

Strain Zn11_249, isolated from the northern area of the SdU, was identified as described in Section 2.7, showing 99.4% similarity to the 16S rRNA gene of *Halomonas titanicae* BH1 (accession number: PRJNA169611) [19], whose genus has recently been renamed *Vreelandella* [8].

### 3.2. Bacterial Growth, EPS Optimization in Three Different Media, and Scanning Electron Microscopy (SEM)

**Figure 1:**
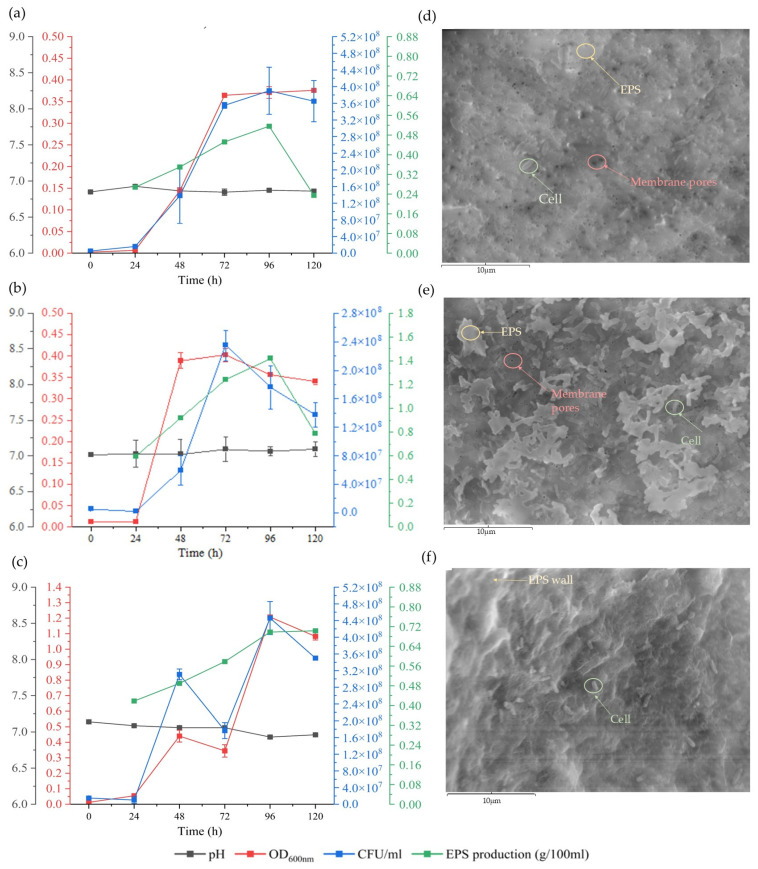
Cell growth, pH values, EPS optimization, and SEM images of *V. titanicae* Zn11_249. (**a**–**c**): Bacterial growth, OD_600_ nm (red), CFU (blue), pH (black), and EPS, g/100 mL (green), under three conditions: (**a**) minimal medium, U_1_; (**b**) minimal medium supplemented with NaCl (1 M), U_2_; (**c**) minimal medium supplemented with LiCl (0.3 M), U_3_. (**d**–**f**): SEM microphotographs, (**d**) EPS in medium, U_1_; (**e**) EPS in medium U_2_; (**f**) EPS in medium U_3_. The SEM images highlight the pores of the membrane where the samples were fixed (red), cells (green), and EPS (yellow).

The following differences in maximum growth values verified by CFU/mL were observed across the different media used and at different time points: U_1_ (3.9 × 10^8^ CFU/mL, 96 h/control), U_2_ (2.25 × 10^8^ CFU/mL, 72 h/kosmotropic), and U_3_ (4.4 × 10^8^ CFU/mL, 96 h/chaotropic), respectively. No acidification of the medium occurred throughout the process (pH 7) (Figure 1). *V. titanicae* Zn11_249 showed a higher CFU/mL than the strain *Halomonas titanicae* MCCC 1A07468 (7 × 10^7^ cells/mL, 72 h), using citrate as a carbon source [37].

The similar bacterial growth shown in U_1_ and U_2_ is likely due to the efficient metabolic performance of *V. titanicae* Zn11_249, which does not require the stimulation of osmolyte synthesis [38,39].

The increased bacterial growth shown in U_3_ corresponds to a metabolic adaptation of *V. titanicae* Zn11_249 to the presence of a chaotropic agent (LiCl) in the medium, leading to a reorganization of its metabolic activity. This could be due to specific stress-adaptation strategies evolved by *V. titanicae* Zn11_249 in its native environment, the SdU. Such adaptation may involve the synthesis of osmolytes to protect membranes and macromolecular structures [39]. The presence of these osmoregulators has already been found in bacteria of the same genus, demonstrating their role in the stress tolerance of these organisms [40].

The highest EPS production by *V. titanicae* Zn11_249 took place in the stationary phase at 96 h (Figure 1). Differences in production were observed in the three media used (U_1_ (36 mg/L/control), U_2_ (42 mg/L/kosmotropic), and U_3_ (336 mg/L/chaotropic)), corresponding to the maximum EPS extracted from the culture medium after purification. In all three cases, the production of EPSs by *V. titanicae* Zn11_249 was higher than that obtained in *Halomonas smyrnensis* K2 (33 mg/L) at 96 h; although, notably, in U_3_, it was significantly higher (336 mg/L), despite a lower NaCl concentration (0.86 M) and a highly enriched culture medium (yeast extract, peptone, and glucose) [12,41]. Moreover, it is higher than *Halomonas ventosae* Al16 (290 mg/L) at 100 h in MY complex medium supplemented with 7.5% (*w*/*v*) marine salts [42]. This would demonstrate that the carbon/nitrogen source has a significant influence in EPS production. This was the case with *Bacillus xiamensis* RT6, where the use of different carbon/nitrogen sources (casein peptone, soybean peptone, glucose) significantly influenced EPS production [33].

On the other hand, in U_1_ (36 mg/L) and U_2_ (42 mg/L), strain Zn11_249 showed minor differences in EPS production. This probably reflects the baseline metabolic activity of the strain under these conditions [38,39].

In the case of U_3_ (336 mg/L) medium, the differences were notable. *V. titanicae* Zn11_249 showed high EPS production in the presence of 0.3 M LiCl. The significant production of EPS in this environment is of industrial interest. This overproduction of EPS might be the physiological reaction as a protective response to the high concentration of this kosmotropic salt [43]. This adaptation strategy has already been described in xerotolerant bacteria [38,44].

To corroborate these results, SEM microscopy was performed (Figure 1). The results showed that, for U_1_ and U_2_, (Figure 1d and Figure 1e, respectively), the observed EPSs were very similar, with relatively sparse distribution. However, in the case of U3 (Figure 1f), a greater presence of EPS was observed, forming a denser and more extensive layer without perceiving the pores of the membrane where the samples were fixed. The EPS could have acted as a protective barrier, allowing for greater bacterial growth in this medium.

### 3.3. Compositional Analysis and Characterisation of EPSs Exopolymer

#### 3.3.1. Molecular Weight Determination of EPSs (EPS_U1_, EPS_U2_, and EPS_U3_) Obtained in Three Different Media

**Figure 2:**
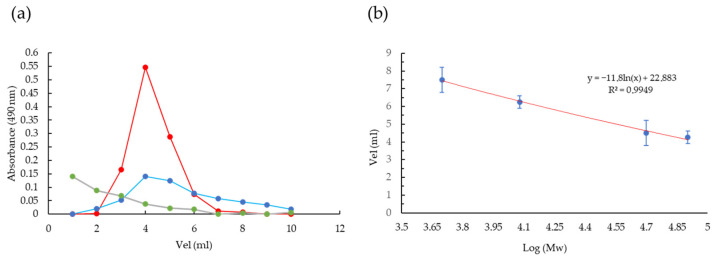
Molecular weight estimation for EPS_U1_, EPS_U2_, and EPS_U3_. (**a**) Elution curve of EPS_U1_ (red), EPS_U2_ (blue), and EPS_U3_ (green) by Sephadex G−100 gel filtration and analysed by the Phenol sulfuric assay. (**b**) Standard curve of the relative molecular weight (Mw).

The estimated molecular weight of the purified EPS was calculated from the calibration curve formula of dextran standards (Figure 2b). The estimated molecular weight for EPS_U1_ (basic medium as control) and EPS_U2_ (kosmotropic medium) was approximately 3.9 × 10^4^ Da, and for EPS_U3_ (chaotropic medium), it was approximately 5.89 × 10^4^ Da (Figure 2b). In all cases, the EPSs presented a molar mass characteristic of the reported polymers (between 4 × 10^4^ and 6 × 10^6^ Da), confirming its typical molecular profile [33,45]. Similar molecular weight values were found in the heteropolysaccharide (mannose and glucose) obtained by *Halomonas almeriensis* M8T in a saline medium supplemented with glucose, yeast extract, and peptone [7] and by *Halomonas saliphila* LCB169T in a saline medium supplemented with sucrose, yeast extract, and peptone [46].

#### 3.3.2. GC-MS Analysis of EPSs (EPS_U1_, EPS_U2_, and EPS_U3_) in Three Different Media

**Figure 3:**
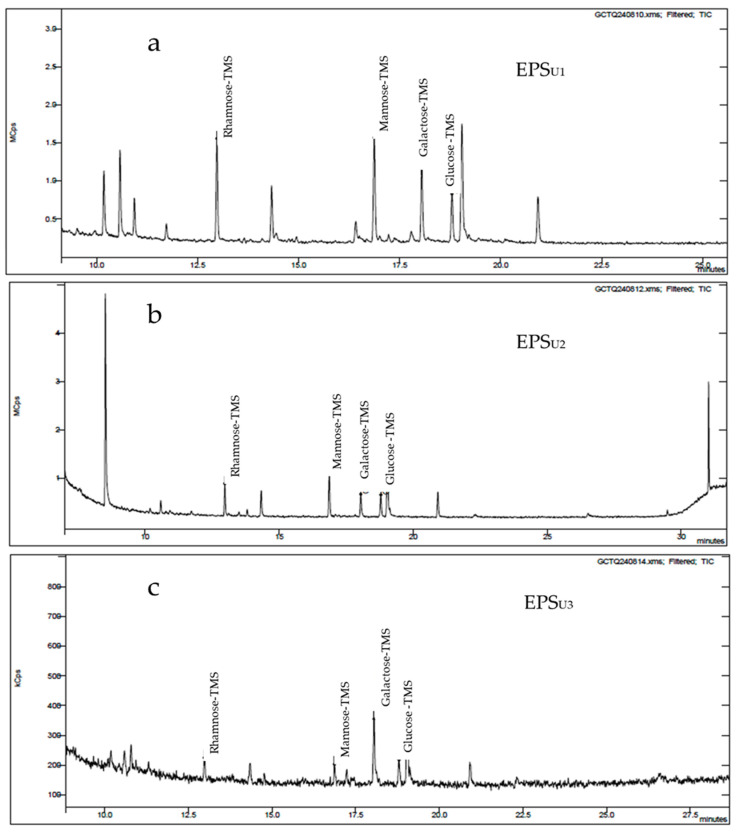
Gas chromatography (GC-MS) analysis of the EPSs of *Vreelandella titanicae* Zn11_249. (**a**) GC-MS analysis of EPS_U1_; (**b**) GC-MS analysis of EPS_U2_; (**c**) GC-MS analysis of EPS_U3_.

Gas chromatography (GC-MS) analysis of the EPSs (EPS_U1_, EPS_U2_, and EPS_U3_) produced by *Vreelandella titanicae* Zn11_249 (Figure 3) showed four peaks, identified as the monosaccharides glucose (α-glucose, β-glucose), β-mannose, α-galactose, and α-rhamnose. These peaks exhibited a similar heteropolysaccharide pattern in the three EPSs analysed. However, the molar ratio [%] of the monosaccharides (mannose, galactose, rhamnose, glucose, respectively) that made up the polymer varied depending on the EPS, as follows: 40/25/25/10 for EPS_U1_, 10/30/30/30 for EPS_U2_, and 25/25/25/25 for EPS_U3_ (Table 1 and Figure 3a, Figure 3b, and Figure 3c respectively). In both media, chaotropic and kosmotropic, a lower ratio of mannose monomers and a higher ratio of glucose monomers were observed. However, none of the media used produced significant changes in the ratios of galactose and rhamnose monomers.

These results indicate that the monomers that made up the EPSs (EPS_U1_, EPS_U2_, and EPS_U3_) of *Vreelandella titanicae* Zn11_249 had a very similar composition within the genus *Halomonas* [47,48]. This is the case for *Halomonas elongata* S6, with the presence of mannose, rhamnose, and glucose, respectively, in a saline medium with the presence of glucose. They had molar ratios of 30.5, 23, and 12, respectively, very similar to that obtained in EPS_U1_ by *V. titanicae* Zn11_249 [11]. This corroborated that the use of the different media employed had a direct influence on the molar ratio of the monomers that made up the EPSs of *V. titanicae* Zn11_249. This could affect the biotechnological activity of the polymers obtained [11,49].

#### 3.3.3. ATR–FTIR Analysis of EPSs

**Figure 4:**
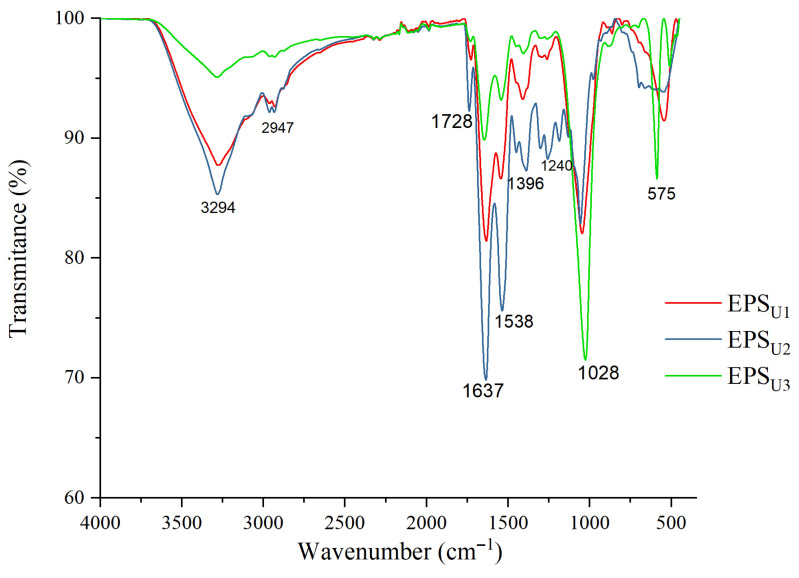
ATR–FTIR analysis of EPS_U1_ (red), EPS_U2_ (blue), and EPS_U3_ (green) produced by *V. titanicae* Zn11_249.

The analysis of the ATR–FTIR spectra (4000 cm^−1^ and 400 cm^−1^) of the EPSs (EPS_U1_, EPS_U2_ and EPS_U3_) produced by *V. titanicae* Zn11_249 is shown in Figure 4. In all three purified samples, the spectrum is the same. The stretching vibration of the hydroxyl group (-OH) had a large peak at 3294 cm^−1^ and a weak stretching band at 2947 cm^−1^ (C=H) [50]. The peak at 1728 cm^−1^ corresponded to the aldehyde functional group (-CHO) [20]. In the case of the peak at 1637 cm^−1^, it could be pointed out as characteristic of the C=O vibration [51]. The peaks at 1538 cm^−1^ and 1396 cm^−1^ were associated with the C-O functional groups, respectively [52]. The peaks at 1240 cm^−1^ and 1028 cm^−1^ corresponded to the glycosidic bond (C-O-C) characteristic of polysaccharides [53]. The region between 1000 cm^−1^ and 500 cm^−1^ could be considered unique to each molecule [33]. The functional groups of the EPSs (EPS_U1_, EPS_U2_, and EPS_U3_) produced by *V. titanicae* Zn11_249 remained unchanged in the three media tested. These functional groups showed similarities with the functional groups of the EPSs of halophilic microorganisms. This was the case for the *Halomonas saliphila* LCB169T strain [46], which also produced a heteropolysaccharide composed of mannose, glucose, and galactose.

#### 3.3.4. Characterisation of the Thermal Properties of EPSs

**Figure 5:**
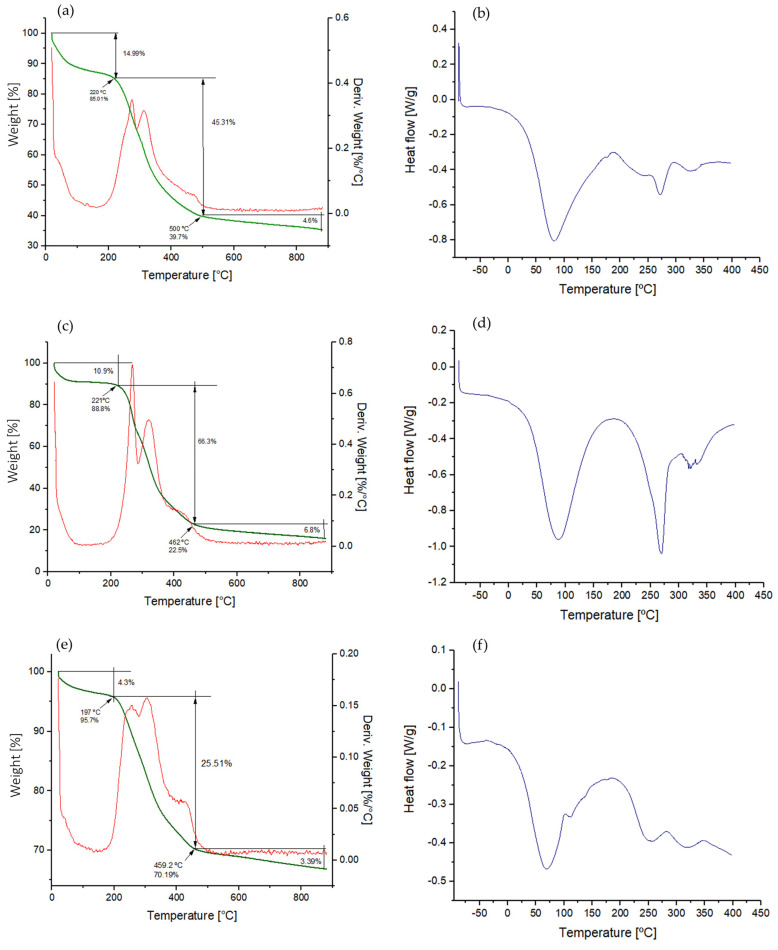
Thermogravimetric analysis (TGA) and differential scanning calorimetry analysis (DSC). Thermogravimetric (TGA) analysis of EPS_U1_ (**a**), EPS_U2_ (**c**), and EPS_U3_ (**e**). Differential scanning calorimetry (DSC) analysis of EPS_U1_ (**b**), EPS_U2_ (**d**), and EPS_U3_ (**f**).

The characterisation of the thermal properties of EPSs (EPS_U1_, EPS_U2_, and EPS_U3_) of *V. titanicae* Zn11_249 was carried out by thermogravimetric analysis (TGA), as shown in Table 2 and Figure 5. The EPSs experienced two states of weight loss as a function of temperature increase. The first stage with an initial weight loss was observed between 20 °C and 250 °C, with a weight loss of 14.99% for EPS_U1_ (Figure 5a), 10.90% for EPS_U2_ (Figure 5c), and 4.30% for EPS_U3_ (Figure 5e), typical of moisture loss, which could be due to water interacting with the functional groups of the EPSs [54].

The second stage was observed between 250 and 500 °C, which presented a gradual weight loss of approximately 45.31% for EPS_U1_ (Table 2 and Figure 5a), 66.30% for EPS_U2_ (Figure 5c), and 25.51% for EPS_U3_ (Figure 5e), reaching stability from this point onwards. These values indicate a major degradation stage between 250 and 500 °C, after which the EPSs reach thermal stability. U_1_ presented a behaviour similar to the thermostable EPS from *Lactobacillus plantarum* HY (67.56% for 200–450 °C) [55].

The thermal behaviour of three *V. titanicae* Zn11_249 EPSs was analysed by differential scanning calorimetry (DSC). The thermograms (Table 2 and Figure 5) showed two melting peaks in all cases for each of the analysed EPSs, as follows: EPS_U1_ (88.76 °C and 272.09 °C), EPS_U2_ (88.76 °C and 270 °C), and EPS_U3_ (63.83 °C and 253.25 °C), as shown in Figure 5b, Figure 5d and Figure 5f, respectively. Similar results were obtained for *Halomonas* sp. S19 (60 °C and 287 °C) [56]. In contrast, EPSs from *Bacillus xiamensis* RT6 generally exhibited lower melting temperatures, reflecting reduced thermal stability [33]. These results demonstrate that the EPSs from *V. titanicae* Zn11_249 exhibit high thermal stability, an important property for potential applications in biotechnology, including the pharmaceutical and food industries [57].

#### 3.3.5. Genomic Analysis of EPS Pathways by *Vreelandella titanicae* Zn11_249

**Figure 6:**
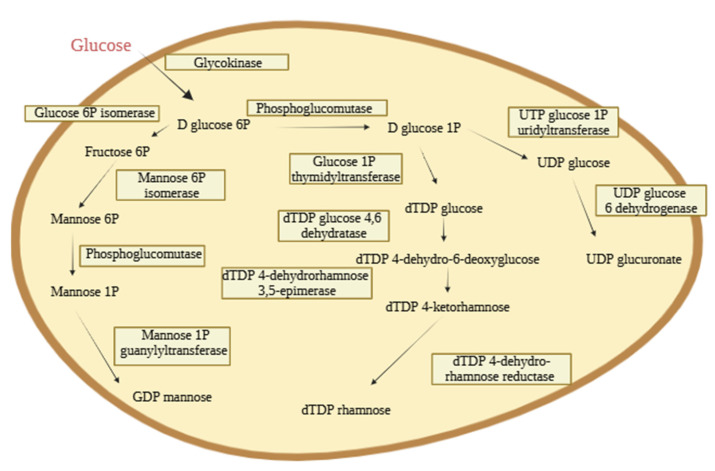
Proposed EPS precursor synthesis pathway using *Vreelandella titanicae* Zn11_249 genome annotation. The proposed synthesis route of precursors in saline medium supplemented with glucose as a carbon and energy source.

The genomic characterisation of *V. titanicae* Zn11_249 (Figure 6) was carried out by bioinformatics tools to identify the genes related to the biosynthesis of EPS using the assembled genome deposited by us in ENA/NCBI (CAYELD010000000/PRJEB81508). The presence of genes involved in the production, export, and structural modification of these biopolymers and their possible monomers was confirmed. For this purpose, the possible synthesis pathways were analysed using glucose as the only carbon source (Section 3.2.). Two possible routes were found, which confirmed the presence of genes responsible for the biosynthesis of EPS by *V. titanicae* Zn11_249 containing rhamnose, mannose, and glucose monomers. The possible biosynthesis pathway of the galactose monomer was not found in the bioinformatic analysis. However, the bacterium is clearly able to incorporate galactose into its EPSs, as confirmed by GC-MS analysis. This suggests that alternative enzymatic routes or non-canonical pathways may exist in *V. titanicae* Zn11_249 to synthesise or activate galactose for EPS biosynthesis.

Firstly, there is the route responsible for the synthesis of two dinucleotide diphosphate sugars, dTDP fructose and dTDP rhamnose. The intracellular pathway of these products begins with the phosphorylation of glucose by a glycokinase (EHLJMEHL_02835) generating D glucose 6P. D glucose 6P is transformed into D glucose 1P by a phosphoglucomutase (EHLJMEHL_04988; EHLJMEHL_03206) which is subsequently used by a thymidyltransferase (EHLJMEHL_04812) to convert it into dTDP glucose. This dTDP glucose is dehydrated to form dTDP 4-dehydro-6-deoxyglucose using a dTDP glucose 4.6 dehydratase (EHLJMEHL_04810).

Finally, dTDP 4-dehydro-6-deoxyglucose is transformed into dTDP 4-ketorhamnose using a dTDP 4-dehydrorhamnose 3,5-epimerase (EHLJMEHL_03653), for a dTDP 4-dehydro-rhamnose reductase (EHLJMEHL_04811) to transform it into dTDP rhamnose. For the synthesis of dTDP fructose, D-glucose 6P, generated by glycokinase, is transformed into fructose 5P through a glucose 6P isomerase (EHLJMEHL_01864; EHLJMEHL_02791) (Figure 6) [58].

The other possible route annotated within the genome of *V. titanicae* Zn11_249 is in charge of the synthesis of UDP glucuronate and GDP mannose from glucose, previously described in *Halomonas malpeensis* YU-PRIM-29T (Figure 6) [48]. Subsequently, a mannose 6P isomerase transforms it into D-mannose 6P. Finally, this is transformed into GDP mannose thanks to a mannose 1P guanylyltransferase (EHLJMEHL_04822) [48].

The genome of *V. titanicae* Zn11_249 presents the genes required to produce fructose and glucuronate. Nevertheless, the lack of these compounds in the composition of the EPSs produced in this study may be due to the use of glucose as the sole carbon source [33].

Genes responsible for the expression of ABC transporter proteins KpsM (EHLJMEHL_04513) and OPX such as KpsD (EHLJMEHL_04514) were also identified, but none were responsible for the Wzx/Wzy-dependent pathway. Therefore, we can hypothesise that the ABC-dependent pathway is the system used by *V. titanicae* Zn11_249 for the secretion of EPS to the cell exterior. Strain Zn11_249 was found to possess a complete pathway for the production of a heteropolysaccharide, previously described in *Halomonas desertis* G11 [58].

### 3.4. Biotechnological Applications

#### 3.4.1. Antioxidant Activity Tests of EPSs

**Figure 7:**
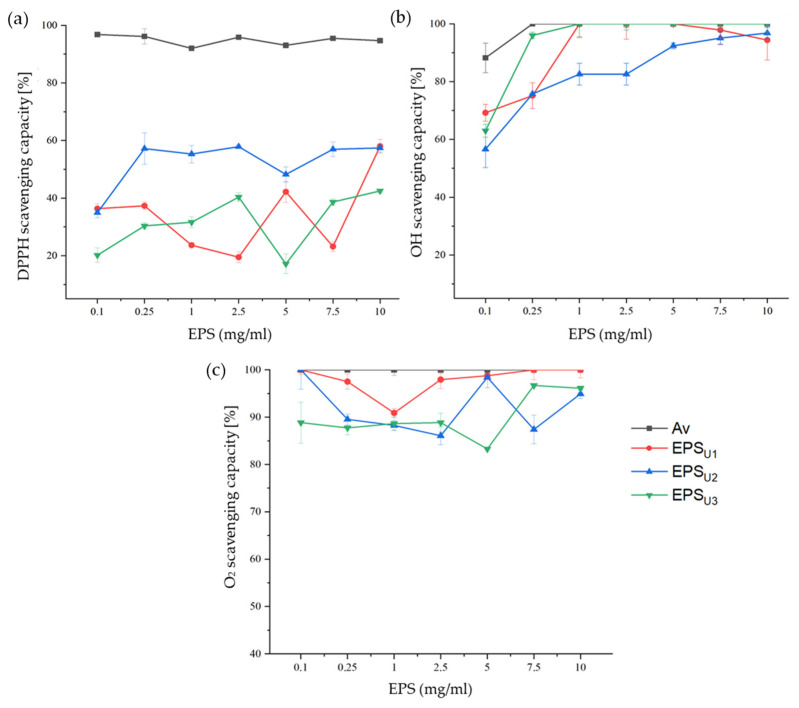
In vitro antioxidant activity with different concentrations of EPSs. (**a**) Free radical DPPH•; (**b**) free radical •OH; (**c**) superoxide radical O_2_−•. L-ascorbic acid (Av) (black) was used as a positive control along with EPS_U1_, EPS_U2_, and EPS_U3_.

The antioxidant properties were evaluated using the following three types of colorimetric assays: 1-diphenyl-2-picryl- hydrazyl radical (DPPH•), the hydroxyl radical (•OH), and the superoxide anion (O_2_−•) (Figure 7). This activity was measured at different concentrations in the range of 0.1–10 mg/mL of the different EPSs. L-ascorbic acid (Av) was used as a positive control.

The antioxidant capacity of the free radical DPPH• of the EPSs (EPS_U1_, EPS_U2_, and EPS_U3_) is represented in Figure 7a. The maximum antioxidant activity for this radical was, for EPS_U1_ (58.0%), EPS_U2_ (57.44%), and EPS_U3_ (42.54%), found at a concentration of 10 mg/mL. This activity is probably due to the presence of hydroxyl functional groups contained in the polymers. These hydroxyl groups could be able to neutralise the oxidizing action of free radicals [33,59]. This was the case with other halotolerant bacteria, such as *Bacillus subtilis* LR-1 (56.00%), at a concentration of 10 mg/mL [60], where hydroxyl functional groups played a relevant role in this activity.

The hydroxyl radical antioxidant capacity of EPSs is shown in Figure 7b. EPS_U1_ (100%) and EPS_U3_ (100%) reached the maximum activity at a concentration of 1 mg/mL. In the case of EPS_U2_ (97.91%), the maximum activity was reached at a concentration of 10 mg/mL. The antioxidant activity of *V. titanicae* Zn11_249 EPSs was higher compared to *Halolactibacillus miurensis* T7, which presented a free radical •OH antioxidant activity of 61% at 3.2 mg/mL of EPS [61]. This activity was also higher than that of other genera of halotolerant bacteria, such as the plant growth promoting endophyte *Glutamicibacter halophytocola* KLBMP 5180, with an activity of 60.81% at a concentration of 0.8 g/L [62]. The higher antioxidant capacity of *V. titanicae* Zn11_249 EPSs could be attributed to the presence of functional groups of donating electrons to neutralise the hydroxyl radical [33].

The superoxide anion antioxidant capacity of EPSs (EPS_U1_, EPS_U2_, and EPS_U3_) is shown in Figure 7c. The highest antioxidant activities against this radical were obtained, for EPS_U1_ (100%), EPS_U2_ (100%), and EPS_U3_ (89.00%), at a concentration of 0.1 mg/mL. This activity is higher than the antioxidant capacity reported for marine *Pseudomonas* PF-6, with 80% at a concentration of 0.1 mg/mL [63]. This high superoxide anion scavenging capacity could be due to the chemical characteristics of EPSs, such as their monomer composition [64]. Additionally, the composition of the culture medium could have influenced the antioxidant activity of *Vreelandella titanicae* Zn11_249 exopolysaccharides by modifying their electronic structure and, consequently, their reactivity to reactive oxygen species [65,66].

Collectively, these results demonstrate that EPSs produced by *V. titanicae* Zn11_249 possess strong non-enzymatic antioxidant properties. Given these characteristics, these EPSs (EPS_U1_, EPS_U2_, and EPS_U3_) have significant potential for industrial applications, particularly as natural antioxidants in the cosmetic, pharmaceutical, and food industries. Their ability to scavenge multiple reactive oxygen species suggests that they could serve as protective agents in formulations aimed at preventing oxidative damage, extending shelf life, and promoting health benefits [67]. Scaling production with high-volume bioreactors and optimised culture conditions could increase EPS yield, enhancing industrial viability and sustainability, as seen in other high-value bioprocesses [68].

#### 3.4.2. Biocompatibility Studies and Antioxidant Ability at Cellular Level of EPSs (EPS_U1_, EPS_U2_, and EPS_U3_) in Three Different Media

**Figure 8:**
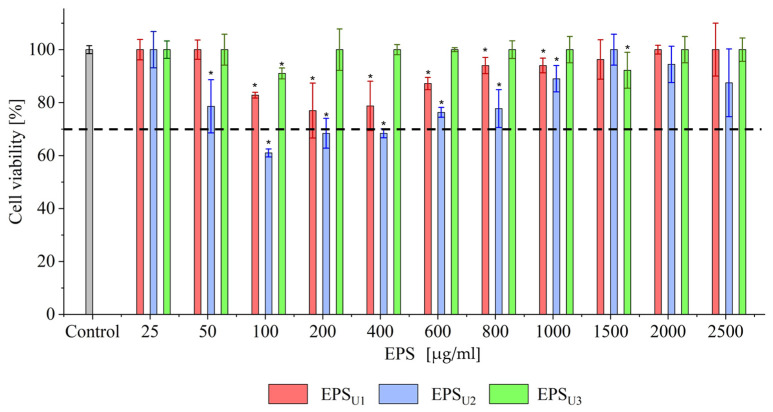
Cell viability of HeLa cells [%] against different EPS concentrations. Cell viability is represented with a negative control without EPSs (grey) and at concentrations of 0.25, 50, 100, 200, 400, 800, 1000, 1500, 2000, and 2500 μg/mL of the different EPSs in the control minimal medium, U1 (red); minimal medium in the presence of NaCl (1M), U2 (blue); and minimal medium in the presence of LiCl (0.3M), U3 (green). The black line indicates it was established at 70% cell viability according to the ISO 10993-5:2009 standard (UNE-EN ISO 10993-5:2009, International Standardisation Organisation) [36]. * *p* < 0.05.

The biocompatibility of the EPSs (EPS_U1_, EPS_U2_, and EPS_U3_) of *V. titanicae* Zn11_249 was carried out with HeLa cells, as shown in Figure 8. Significant differences were observed for EPS_U2_ and EPS_U3_ with respect to EPS_U1_ (*p* > 0.05). The statistical differences were complemented according to the biocompatibility limit established at 70% cell viability according to the ISO 10993-5:2009 standard [36]. Cell viability analysis showed that EPS_U1_ and EPS _U3_ obtained cell viability values above 80% and 90%, respectively, at all concentrations studied. In the EPS_U2_ case, cell viability values were higher than 70%, except for the tested concentrations of 100 (60.97%), 200 (68.41%), and 400 (68.37%) μg/mL. In the three polymers, no dose-dependent correlation was observed. This was also the case with *Bacillus licheniformis* IDN-EC at concentrations of 400 (85%) and 600 (81%) μg/mL [22,69].

#### 3.4.3. H_2_O_2_-Induced Assay and Effects of EPSs

**Figure 9:**
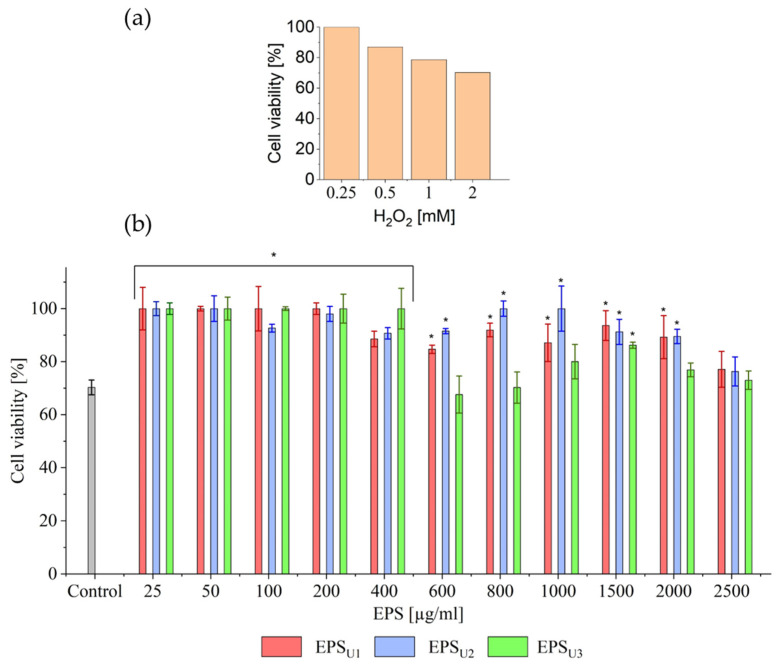
Cell viability of HeLa cells [%] against different concentrations of EPSs. (**a**) HeLa cell viability (%) against oxidative stress at different concentrations of H_2_O_2_. (**b**) Treatment for the protection of HeLa cell viability (%) at 0.25, 50, 100, 200, 400, 800, 1000, 1500, 2000, and 2500 μg/mL of EPS_U1_ (red), EPS_U2_ (blue), and EPS_U3_ (green). As a negative control, cell viability was analysed without the presence of EPS (grey). * *p* < 0.05.

The antioxidant capacity of EPSs (EPS_U1_, EPS_U2_, and EPS_U3_) of *V. titanicae* Zn11_249 at the cellular level was evaluated with HeLa cells at different H_2_O_2_ concentrations (Figure 9a). A 30% decrease in cell viability was observed at the maximum H_2_O_2_ concentration used (2 mM) due to the accumulation of reactive oxygen species (ROS).

To determine the antioxidant effect of EPSs (EPS_U1_, EPS_U2_, and EPS_U3_) of *V. titanicae* Zn11_249 on HeLa cells (Figure 9b), the protective capacity of EPSs against damage caused by H_2_O_2_ (2 mM) was evaluated. The results demonstrated a significant protective effect of all EPSs at concentrations between 25 and 400 μg/mL, with cell viability values greater than 90% significantly different from the control. These results demonstrated improved cell recovery at low concentrations compared to the antioxidant activities of extremophilic bacteria, such as *Bacillus licheniformis* IDN-EC and *Bacillus amyloliquefaciens* RT7 with 90% cell viability at 25 μg/mL [22,52].

This reduction in injury by the EPSs of *V. titanicae* Zn11_249 could be consequence to the stimulation of the antioxidant system by the EPSs, which allowed for a significant increase in cell protection [52,69].

## 4. Conclusions

This work reports the growth, production, characterisation, bioinformatic analysis, cytotoxicity, and biotechnological applications of EPSs produced by the strain *Vreelandella titanicae* Zn11_249, isolated from the Salar de Uyuni, in three different culture media.

Under all three conditions studied, EPS production was significant. However, the most notable production occurred under chaotropic conditions in LiCl (336 mg/L of EPS). This represents a significant improvement compared to other halophilic bacteria of the same genus using defined growth conditions, and it demonstrates a novel and promising approach for industrial EPS production.

In the characterisation of the polymers obtained under the three conditions, no changes in the chemical composition of the EPS monomers were observed. High thermostability was observed, which is an important property for their biotechnological applications. Genomic analysis of *V. titanicae* Zn11_249 identified potential genes responsible for the biosynthesis of EPS containing rhamnose, mannose, and glucose monomers, which agreed with the monomer content of the polymers. This type of genomic analysis implements and corroborates the characterisation of EPS.

In terms of bioactivity, the EPSs exhibited low cytotoxicity in HeLa cells. The EPSs displayed great biotechnological potential as non-enzymatic antioxidants, even superior to those described form other halophilic EPSs. This makes them highly suitable for use in industrial applications such as food, cosmetics, and biomedicine.

The production of EPS from *V. titanicae* Zn11_249 represents a sustainable and promising alternative that contributes to the Sustainable Development Goals (SDGs) by generating safe and environmentally friendly biopolymers for industrial, food, and cosmetic applications. Therefore, this work lays the groundwork for future research into biopolymers with biotechnological potential, produced by bacteria in an unexplored environment such as Salar de Uyuni.

## Figures and Tables

**Table 1:** Monosaccharide composition (molar ratio [%]) of EPSs produced by *Vreelandella titanicae* Zn11_249 analysed by GC-MS. The molar ratios vary depending on the EPS and the medium used, U1, U2, and U3.

EPS	Glucose (Molar Ratio [%])	Mannose (Molar Ratio [%])	Galactose (Molar Ratio [%])	Rhamnose (Molar Ratio [%])
EPS_U1_	10	40	25	25
EPS_U2_	30	10	30	30
EPS_U3_	25	25	25	25

**Table 2:** Thermal properties of EPSs (EPS_U1_, EPS_U2_, and EPS_U3_) produced by *Vreelandella titanicae* Zn11_249. TGA shows two weight-loss stages, corresponding to moisture loss (20–250 °C) and major degradation (250–500 °C), after which thermal stability is reached. DSC analysis reveals two melting peaks (Tm1, Tm2) for each EPS.

EPS	Initial Weight Loss 20–250 °C (%)	Major Weight Loss 250–500 °C (%)	DSC Tm1 (°C)	DSC Tm2 (°C)
EPS_U1_	14.99	45.31	88.76	272.09
EPS_U2_	10.90	66.30	88.76	270.00
EPS_U3_	4.30	25.51	63.83	253.25

## Data Availability

The original contributions presented in this study are included in the article. Further inquiries can be directed to the corresponding author.
